# Knowledge of women in Poland on the profession and competencies of a midwife

**DOI:** 10.18332/ejm/183910

**Published:** 2024-03-19

**Authors:** Agnieszka Wyrębek, Julia Klimanek, Alicja Misztal, Beata Szlendak, Grażyna Bączek

**Affiliations:** 1 Department of Obstetrics and Gynecology Didactics, Faculty of Health Sciences, Medical University of Warsaw, Warsaw, Poland; 2 Department of Midwifery, Center of Postgraduate Medical Education in Warsaw, Warsaw, Poland

**Keywords:** knowledge, Poland, women, midwife, competencies

## Abstract

**INTRODUCTION:**

The profession of a midwife, despite its long tradition in Poland, is still not widely known in the society. Both in terms of the competencies and roles, it is often confused with other medical professions such as nurses or gynecologists. In this study, we assessed the knowledge of women in Poland on the profession of a midwife. The aim of the research project was to obtain detailed data on the knowledge of women regarding the specific professional competencies of midwives.

**METHODS:**

The study used the method of a cross-sectional survey. A survey of 1134 adult Polish women was conducted. A 20-item questionnaire was developed with fourteen of the questions being based on the midwife’s professional competencies. The study was conducted in 2019 and the questionnaire was distributed through various social groups for Polish women.

**RESULTS:**

Knowledge about the professional competencies of midwives increases proportionally to the level of the education of the respondents, their age and the scope of cooperation with midwives. The most well-known forms of midwifery were those related to lactation education (78.7%) and puerperal care (78.9%). The lowest rates, among other results, were prescribing drugs (23.1%) and collecting samples for cervical cytology (24.4%).

**CONCLUSIONS:**

The profession of a midwife in Poland is insufficiently popularized. Competencies shared with doctors require more dissemination. It is worth paying particular attention to the promotion of the profession in younger age groups, so that women can use their knowledge at subsequent stages of their lives.

## INTRODUCTION

In Poland, the practice of the midwifery profession is regulated by the Act of 1 July 2011, on the Professions of a Nurse and a Midwife^1^. It defines the places of employment, professional competencies, and conditions to be met in order to practice the profession. The midwifery profession in Poland has evolved over the years. Before the 18th century, the oldest women in a family, who helped other women with their experience, were considered midwives. Their knowledge was derived from personal experience and orally passed from generation to generation. The duration of this education has changed multiple times over the years, from several months of training to 2.5–3.0 years of education in midwifery schools. Due to requirements set by the European Union, since 2003, the only way to become a midwife is to complete a 3-year Bachelor’s degree program at a medical university. After graduation, it is possible to obtain a Master’s degree and further academic titles. Continuing postgraduate education in specialties, courses, or postgraduate studies such as an EMBA in healthcare is also an option. Over the years, the competencies of midwives have also changed. For example, the duration of care for a newborn was extended from 9 days to 6 weeks and, currently, up to the end of the second month of life. Since 1 January 2016, the competencies of Polish midwives have been expanded to include the ability to prescribe certain medications and to refer for specific examinations as determined by the Minister of Health’s regulation^1^.

The World Health Organization (WHO) and the National Institute for Health and Care Excellence (NICE) indicated the midwife as the most suitable medical worker for the provision of primary care for a woman and her child. Midwives provide assistance during physiological pregnancy, childbirth, and puerperium, as well as newborn care and preparation for childbirth and parenthood. Such a solution is considered to be the best in the context of the economic availability of medical services for women in primary-care institutions^2,3^.

The aim of this study was to assess the level of knowledge of women in Poland about the profession and competencies of a midwife, with an emphasis on which competencies and rights of a midwife were the most and which were the least known among Polish women.

## METHODS

### Study design, sample and setting

A cross-sectional survey using a questionnaire was conducted to assess the competencies of midwives in Poland. A convenience sample was used with the questionnaire distributed through social media platforms (Facebook) within groups associated with various cities in Poland, in February 2019. The inclusion criteria of the study were female sex and aged ≥18 years. A total of 1237 responses were collected, with 103 excluded due to incomplete or incorrect responses. The research project obtained approval from the Ethics Committee in Warsaw (Approval number: AKBE 161/17; Date: 5 September 2017). The participants were informed about data collection for scientific purposes, and they provided informed consent, agreeing to the processing of all shared data for the research project.

### Data collection

The questionnaire consisted of 20 questions prepared using Google Forms. The form was divided into two sections. The first section comprised seven questions aimed at determining the respondent’s profile based on age, education level, marital status, place of residence, completing medical education, and experiences related to obstetric and gynecological events in life. The second section included 14 questions directly related to the knowledge about who a midwife was, the respondent’s association with the midwifery profession, and the perceived competencies of midwives.

### Data analysis

Statistical analyses were conducted using IBM SPSS Statistics version 25. The analyses included basic descriptive statistics, frequency analysis, correlation analyses using Pearson’s r and Spearman’s rho coefficients, Student’s t-test for independent samples, and the Kruskal-Wallis test.

## RESULTS

The largest group of respondents (74.6%) included women aged 18–25 years. The majority of the respondents had never been married (74.8%), and over half of them (51.5%) lived in a city with a population of ≥100000 inhabitants. Almost half of the women (49.7%) completed secondary education. Additionally, the respondents were divided into two groups – women with medical (17.2%) and non-medical education (82.8%). Midwifery care had already been provided to 36.6% of the women. Detailed results are presented in Table 1.

**Table 1:** Sociodemographic characteristics of women aged ≥18 years participating in the survey to assess their knowledge on the competencies of midwives, Poland, 2019 (N=1134)

*Characteristics*	*%*	*n*
**Age** (years)		
18–25	74.6	846
26–39	21.8	248
40–61	3.5	40
**Marital status**		
Single (never married) woman	74.8	848
Married	22.9	260
Divorced	2.1	24
Widow	0.2	2
**Residence**		
City (≥100000 inhabitants)	51.5	584
Town (<100000 inhabitants)	26.0	295
Village	22.5	255
**Education level**		
Tertiary	42.9	486
Secondary	49.7	564
Junior high school	4.9	56
Vocational	2.2	25
Primary	0.3	3
**Type of education**		
Medical*	17.2	195
Non-medical	82.8	939

* Medical professions: doctor, midwife, nurse, medical laboratory scientist, pharmacist, paramedic, and physiotherapist.

The respondents were presented with statements referring to selected and only true professional competencies of midwives in Poland and employment opportunities. Over half of the respondents were sure that it was the midwife who provided advice on breastfeeding and supported its course (78.7%), took care of the mother and the newborn by monitoring the postpartum period (78.9%), and educated parents about newborn care (76.4%). A total of 35.2% of them claimed that midwives had appropriate qualifications and competencies to diagnose pregnancy and to provide prenatal care under the National Health Fund or privately (33.5%). Fewer than half of the respondents (46.2%) declared that midwives were independent regarding physiological delivery, and over half of them strongly agreed with the statement that midwives were qualified to attend vertical delivery (60.3%). Respondents also thought that if necessary, during childbirth, midwives may perform episiotomy (42.5%) and the perineal suture (27.9%). Only 19.8% of the respondents were convinced that midwives could refer patients for some diagnostic tests, and 57.3% of women were unaware that midwives could prescribe certain drugs. Detailed results are presented in Table 2.

**Table 2:** Knowledge of women aged ≥18 years participating in the survey on the qualifications and competencies of a midwife, Poland, 2019 (N=1134)

*Midwife role*	*Strongly agree*		*Agree*		*I don’t know*		*Disagree*		*Strongly disagree*	
	*n*	*%*	*n*	*%*	*n*	*%*	*n*	*%*	*n*	*%*
Prepares a woman (couple) for pregnancy, childbirth, and puerperium	780	68.8	208	18.3	82	7.2	54	4.8	10	0.9
Conducts educational and health activities in the area of reproductive health	543	47.9	357	31.5	149	13.1	73	6.4	12	1.1
Is able to diagnose pregnancy	399	35.2	365	32.1	138	12.2	186	16.4	46	4.1
May provide prenatal care in case of physiological pregnancy within the framework of the NHF and private practice	380	33.5	255	22.5	184	16.3	205	18.0	110	9.7
May refer for tests necessary for the early diagnosis of high-risk pregnancy	327	28.8	305	26.9	229	20.2	197	17.4	76	6.7
Is independent when attending a physiological delivery	524	46.2	338	29.8	109	9.6	116	10.2	47	4.2
Performs episiotomy if necessary	482	42.5	285	25.1	201	17.7	118	10.4	48	4.3
Performs perineal suturing after episiotomy	316	27.9	254	22.4	264	23.3	196	17.3	104	9.2
Attends a vertical delivery	684	60.3	295	26.0	119	10.5	28	2.5	8	0.7
Monitors the condition of the fetus using medical equipment	673	59.3	296	26.1	101	8.9	45	4.0	19	1.7
Gives advice on breastfeeding and supports its course	893	78.7	192	16.9	36	3.2	12	1.1	1	0.1
Provides care to the mother and the newborn, and monitors the course of the postpartum period	895	78.9	189	16.7	34	3.0	10	0.9	6	0.5
Provides nursing care to the newborn and educates parents in this area	866	76.4	203	17.9	47	4.1	8	0.7	10	0.9
Recognizes abnormal symptoms in the mother and child and refers them to a physician	701	61.8	305	26.9	89	7.8	28	2.5	11	1.0
Provides obstetric and gynecological care to women	680	60.0	294	25.9	91	8.0	56	4.9	13	1.2
Provides support in childbirth, motherhood, in case of miscarriage, infertility, or gynecological diseases	666	58.7	327	28.8	102	9.0	30	2.7	9	0.8
Prepares women for gynecological procedures and operations	514	45.3	318	28.0	180	15.9	98	8.6	24	2.2
Educates and rehabilitates women after mastectomy	260	22.9	234	20.6	390	34.4	156	13.8	94	8.3
May collect samples for cervical cytology (Pap test)	277	24.4	207	18.2	289	25.5	221	19.5	140	12.4
Visits women and newborns at home after discharge	793	69.9	198	17.5	94	8.3	34	3.0	15	1.3
Works for the prevention of women’s health issues and obstetric pathologies	568	50	342	30.2	171	15.1	42	3.7	11	1.0
May issue a prescription and prescribe certain medications	262	23.1	222	19.6	240	21.2	222	19.6	188	16.5
May issue a referral for specific diagnostic tests	225	19.8	243	21.4	291	25.7	217	19.2	158	13.9
May supervise and manage teams of nurses or midwives	495	43.6	339	29.9	194	17.1	70	6.2	36	3.2
May conduct scientific research	594	52.4	300	26.5	161	14.2	47	4.2	32	2.7

The vast majority of the respondents indicated that the delivery room (98.8%) and the maternity ward (98.6%) were midwives’ workplaces. Only 21% of the women were convinced that midwives were employed in nursing homes. About one-third (29.1%) also indicated administrative and supervisory positions in medical entities as the workplaces of midwives. Detailed results are presented in Table 3.

**Table 3:** Workplaces of midwives according to women aged ≥18 years participating in the survey, Poland, 2019 (N=1134)

*Workplaces*	*Strongly agree*		*Agree*		*I don’t know*		*Disagree*		*Strongly disagree*	
	*n*	*%*	*n*	*%*	*n*	*%*	*n*	*%*	*n*	*%*
Primary healthcare	573	50.5	372	32.8	111	9.8	66	5.8	12	1.1
Delivery room	1032	91.0	88	7.8	7	0.6	6	0.5	1	0.1
Department of pregnancy pathology	839	74.0	239	21.1	41	3.6	13	1.2	2	0.2
Neonatology	583	51.4	244	21.5	254	22.4	46	4.1	7	0.6
Obstetrics	1027	90.6	90	8.0	10	0.9	4	0.3	3	0.2
Gynecology	796	70.2	239	21.1	55	4.9	40	3.5	4	0.3
Department of gynecological oncology	553	48.8	304	26.8	165	14.5	89	7.9	23	2.0
Lactation clinic	783	69.0	265	23.4	65	5.7	18	1.6	3	0.3
Antenatal classes	922	81.3	179	15.8	19	1.7	10	0.9	4	0.3
Fertility treatment clinics/departments	462	40.7	324	28.6	192	16.9	121	10.7	35	3.1
Nursery	367	32.4	311	27.4	208	18.3	178	15.7	70	6.2
Nursing home	240	21.0	331	29.3	266	23.5	192	16.9	105	9.3
Medical university	574	50.6	316	27.9	136	12.0	80	7.0	28	2.5
Administrative and supervisory positions in medical entities	330	29.1	344	30.0	248	22.0	150	13.3	62	5.6

It was examined whether sociodemographic variables and the use of midwifery services in the past correlated with the knowledge about the midwife’s profession and competencies. Statistically significant associations were demonstrated between the knowledge and the age of the respondents, their level of education, place of residence, marital status, and the scope of contact with midwives as part of health services. Detailed data are presented in Table 4.

**Table 4:** List of correlations between variables of women aged ≥18 years participating in the survey and knowledge of the competencies of midwives, Poland, 2019

*Variables*	p*	*Pearson’s r*	*Spearman’s rho*	*Student’s t-test*
Knowledge about midwives increases with age	0.009	0.08	-	-
Knowledge about the profession of midwives increases with the level of education	<0.001	-	0.18	-
Knowledge about the profession of midwives increases with the size of the inhabited town	0.023	-	0.07	-
Married women have a higher level of knowledge about the midwife’s profession and her competencies	<0.001	-	-	3.53
Women with medical education know more about the midwifery profession	<0.001	-	-	15.31
Knowledge about the profession increases with the number of times the respondent used a midwife’s services	0.035	0.06	-	-
The better women evaluate cooperation with a midwife, the more knowledgeable they are about the profession	<0.001	-	-0.19	-
Women who used the services of a midwife have more knowledge about the profession and midwife’s competencies	<0.001	-	-	6.85
Women who previously used the services of a midwife other than in the field of perinatal issues obtained significantly lower results in terms of knowledge compared to the group that used both perinatal and other midwifery services	0.013	-	-	-

* Statistical significance at p<0.05.

## DISCUSSION

The present study showed that women’s knowledge about the profession and midwife competencies increased with the age of the respondents. It may be correlated with greater life experience and more frequent use of midwifery care, not only in connection with motherhood but also in the field of gynecological care and contact with people who have used midwifery care. This was confirmed by the present research, which revealed that knowledge about the profession increased significantly in the respondents, along with the frequency and diversity of midwifery services used.

It was demonstrated that married women had a significantly higher level of knowledge in the studied area. This may be related to more frequent use of the services offered by midwives in connection with family planning, attending antenatal classes, childbirth, and puerperium.

According to the present study, increasing knowledge about the profession and competencies of a midwife was noted, along with an increase in the level of the respondents’ education. Raghupathi et al.^4^ confirmed that, statistically, people with higher education took better care of their health and had greater awareness concerning health promotion.

The present study confirmed that the place of residence exerted an effect on the level of knowledge of the midwifery profession, which increased with the size of the city where the respondents lived. Cities are characterized by a higher number of professionally active midwives and a higher number of medical and specialist centers. Large agglomerations provide a greater and easier opportunity to use various forms of midwifery services in public and private sectors, e.g. services of International Certified Lactation Consultants IBCLC and antenatal classes^5-7^.

Every pregnant woman in Poland between 21 and 26 gestational weeks should be referred to a community midwife at a Primary Health Care Clinic for antenatal education. As the present study showed, this contributed to the respondents’ high knowledge of the educational role of midwives. Prenatal education provided by a midwife is also appreciated among women in other countries, which was confirmed by other authors, e.g. Liu et al.^8^. In order to maintain the continuity of post-discharge medical care, women and their newborns are again referred to a midwife tο be provided care as part of educational and follow-up home visits. During such visits, the midwife may inform the patient about the diversity of services offered, which may constitute an element of the promotion of the profession^1,8,9^.

Regarding all the competencies of a midwife in Poland (some of them listed in Table 2), women may mainly benefit from midwives’ services from the stage of pregnancy planning to the end of the puerperium, including three follow-up visits to a gynecologist^9^. A midwife, being the main specialist in the model of care for women and their newborns, is involved in the standard of medical care in some countries, e.g. in the United Kingdom^2^. To date, it has not become the general practice in Poland because, as research shows, the possibility of obtaining antenatal care from a midwife is not widely known to Polish women. Leja-Szpak et al.^10^ reported that 31% of their respondents would consider resigning from antenatal care offered by a physician in favor of a midwife under the National Health Fund (NHF) due to the individual approach of midwives to patients, their greater involvement, time spent and greater focus on the naturalness of the processes occurring^10^. Research by other authors and health-related organizations indicated that such a model of care was the most desirable. It prevented excessive medicalization and was the most recommended in the world for outpatient and inpatient care^2,3,8^. Data collected by Edmonds et al.^2^ indicated that midwifery care was associated with a number of positive results. It did not increase the risk of negative effects for either the mother or the child; it reduced the number of unnecessary medical interventions and increased patient satisfaction. The National Institute for Health and Care Excellence (Nice) in the United Kingdom indicated a midwife as the most suitable person to assist in childbirth for women at a low perinatal risk^2^.

The present study showed the legitimacy of promoting the participation of midwives in the care of women among the public due to the insufficient knowledge of the respondents in this area. A study by Edmonds et al.^2^ indicated that high-quality midwifery care improved over 50 health outcomes in various areas, including gynecology and oncology^2^. Midwives in Poland are authorized to collect material for cervical cytology (Pap smear). Numerous legal regulations and the opinion of the Province Consultant in the field of Gynecological and Obstetric Nursing for the Mazowieckie Province clearly indicate that midwives may perform cervical cytology without additional training^11^. The completion of an additional course is only required when the examination is performed as part of the National Cervical Cancer Prevention Program. The Cervical Cancer Prevention Clinic at the Oncology Center – Maria Skłodowska-Curie Institute in Warsaw, may be a good model for the collection of cervical cytology samples, where cytology smears are mainly performed by midwives^1,12,13^.

The present study revealed a low level of the respondents’ knowledge concerning the possibility of issuing prescriptions and prescribing certain drugs by a midwife. According to the National Health Fund, the number of nurses and midwives issuing prescriptions is constantly growing^14-16^. Positive international experiences in this area date back several decades and reduce inequalities in access to fast, professional healthcare. Research conducted in Australia demonstrated a growing tendency for midwives to issue prescriptions, e.g. for antibiotics. The correctness of their selection in accordance with therapeutic guidelines was confirmed. Midwives were found to select narrowspectrum antibiotics more often than physicians, which is important in global strategies to counteract growing antibiotic resistance^17^.

As regards the present study, the respondents were aware of the possibility of a midwife conducting scientific research and supervising and managing teams of nurses or midwives^1^. An increase in the activity of midwives in this area has been noted in Europe in recent years. It is important to signal scientific and managerial opportunities to the public and other healthcare workers^18^. This will help build a positive image of the midwife in the fight against the misidentification of midwives as ‘intermediate-level personnel’. The Regulation of the Minister of Labour and Social Policy on the Classification of Professions and Specialties classifies midwives into the category of ‘specialists’ regardless of whether they have reached the highest education level^19^. A study by Mivsek et al.^20^ confirmed that the awareness of the rank of the profession had a positive impact on cooperation and relations between professions.

A high rate of medicalization in obstetrics, e.g. an increased percentage of cesarean sections, poses a threat to the professional position of midwives because medical care plays a greater role in such cases^21^. Chakrabory et al.^22^ reported an alarming shortage of midwives whose services were crucial to meeting the health needs of women and children. An increased number of midwives was found to contribute to the reduction of perinatal mortality.

Attracting candidates to the profession requires the effective promotion of the midwifery profession, its competencies, role, and opportunities. Increasing social awareness and the global unification of educational standards, competencies, and professional autonomy is essential. This should be a priority to facilitate the migration of midwives to areas with limited access to professional care, which will make it possible to bridge gaps and inequalities in access to midwifery care. Increasing the share of professional midwifery in the care of a woman at every stage of her life and of a newborn is crucial for the implementation of the Sustainable Development Goals (SDGs) and 2030 WHO guidelines, and will allow the use of healthcare resources in an economic, ergonomic and holistic way^2,23,24^. It is advisable for the healthcare system in Poland to use the above guidelines when planning changes in midwifery education, practice, and subsequent scientific research.

### Strengths and limitations

Certainly, the study of women’s knowledge about midwives’ competencies requires deepening and continuation. The size of the group may be considered a strength of the present study, but obtaining data through social media reduces the participation of older people. The limitations of the study include the inability to generalize the study results to the entire population of Poland, due to the convenience sample of adults. The selection method used via social media increased the participation of young people in the study, which may contribute to selection bias. Therefore, systematic research comprising the participation of different age groups is necessary.

## CONCLUSIONS

The most well-known competencies of a midwife included those related to education, pregnancy, childbirth, and puerperium. The awareness of the midwifery profession and its competencies is influenced by sociodemographic factors such as education or the place of residence. Therefore, efforts should be made to equalize these differences by promoting knowledge about the midwifery profession tailored to the specific needs of various social groups. This will enable all patients to make informed decisions and access high-quality midwifery services at various stages of life. Moreover, initiatives aimed at promoting the role and skills of midwives are mandated by the Act of 1 July 2011, on the Professions of a Nurse and a Midwife. Complete use of the professional rights of midwives will allow the reasonable use of healthcare resources and facilitate patients’ access to professional medical services.

## Data Availability

The data supporting this research are available from the authors on reasonable request.
